# Stroke Prevalence and Risk Factors in Rural Communities Within a Resource-Constrained South Asian Setting: Population-Based Study of 1.3 Million Individuals

**DOI:** 10.2196/46122

**Published:** 2025-12-19

**Authors:** Farhana Sarker, Rony Chowdhury Ripan, Moinul H Chowdhury, AKM Nazmul Islam, Mashiar Rahman, Shariful Islam, Shumit Saha, Khondaker A Mamun

**Affiliations:** 1 Department of Computer Science and Engineering, Independent University Bangladesh (IUB), Dhaka, Bangladesh; 2 Center for Computational and Data Sciences, Independent University Bangladesh (IUB), Dhaka, Bangladesh; 3 CMED Health (A Digital Health inclusion initiatives for UHC), Dhaka, Bangladesh; 4 Palli Karma-Sahayak Foundation, Dhaka, Bangladesh; 5 Department of Nutritional Sciences, Texas Tech University, Lubbock, TX, USA; 6 School of Applied Computational Sciences, Meharry Medical College, Nashville, TN, United States; 7 Advanced Intelligent Multidisciplinary Systems Lab, Institute of Research, Innovation, Incubation and Commercialization (IRIIC), United International University, United City, Madani Ave, Dhaka, 1212, Bangladesh, 880 1776534220; 8 Department of Computer Science and Engineering, United International University, Dhaka, Bangladesh

**Keywords:** stroke, prevalence, Bangladesh, community health workers, survey, health workers, rural areas, risk factor, hypertension, diabetes

## Abstract

**Background:**

Stroke is a leading cause of death and long-term disability worldwide, with an estimated 6.2 million deaths each year. In Bangladesh, data on stroke prevalence and risk factors in rural areas are limited, making it difficult to develop effective early prevention and intervention programs.

**Objective:**

This study aimed (1) to present the prevalence of stroke in a rural community in Bangladesh and (2) to identify and associate various stroke risk factors.

**Methods:**

Data collection was done by community health workers, as a part of the “Enriched Sastho” program of the Palli Karma Sahayak Foundation, Bangladesh. Community health workers received 2 weeks of training to ensure data quality. The presence of stroke was determined by a binary survey question, with a history of stroke=1 and absence=0. The prevalence of stroke per 1000 people was examined along with the 95% CI. In addition, the association of stroke risk predictors was calculated using multivariate logistic regression and presented in crude odds ratio (OR) and adjusted OR along with 95% CI.

**Results:**

The study analyzed data from 1,341,589 individuals, with an average (SD) age of 29.23 (19.05) years. The overall stroke prevalence was found to be 1.07 per 1000 people, with a higher prevalence in male participants and increasing with age. The highest stroke prevalence was observed in the Khulna division (OR 1.881, 95% CI 1.671‐2.117), and the least in the Rangpur division (OR 0.677, 95% CI 0.576‐0.795). Individuals aged 65‐79 years were at a higher risk of having a stroke than other age groups (crude OR 9.883 and adjusted OR 9.728 [adjusted for sex]). In addition, male participants were at greater risk of having a stroke than female participants were (crude OR 1.565 and adjusted OR 1.469 [adjusted for age]).

**Conclusions:**

The study emphasizes the need for early prevention and intervention programs for stroke in rural Bangladesh and the importance of managing hypertension and diabetes to reduce stroke risk.

## Introduction

Stroke, heart attack, irregular heart rhythms, and other cardiovascular diseases (CVDs) are the leading causes of death and long-term disability globally [1]. An estimated 6.2 million people die each year from CVDs alone, which is 11% of all global fatalities [2]. In 2019, approximately 17.9 million individuals lost their lives to CVDs, accounting for about 32% of total global deaths [3]. Stroke is the leading cause of death from CVDs [4]. There were 15.2 million fatalities from ischemic heart disease and stroke in 2015 [5]. Khan et al [6] analyzed data from 1990 to 2017 and reported 9 million deaths were caused by ischemic heart disease globally. According to the World Health Organization, stroke was the second greatest cause of mortality and the sixth biggest cause of disability-adjusted life years (DALYs) loss worldwide in 2002 [7]. There has been a steady rise in the number of survivors with stroke and the number of stroke-related DALYs. Worldwide, there are over 62.1 million survivors with stroke [8], with 36.2 percent of those survivors left with a significant disability up to 5 years following a stroke [9]. In 2019, stroke continued to be the second most common cause of death worldwide, accounting for 11.6% of all deaths [10]. It also ranked as the third leading cause of both death and disability combined, representing 5.7% of total DALYs [10].

While 75% of stroke-related fatalities occur in low- and middle-income countries [11], there has been less research conducted in these areas. Bangladesh is a low- and middle-income country with a population of 162.2 million people, the vast majority of whom live in rural communities (74%) [12]. The government spends only US $123 per person a year on health care [13]. It is estimated that one-third of Bangladeshis are aged <35 years. There are currently 14.6 million people aged >60 years in Bangladesh, and that number is expected to rise to 55.7 million by the year 2061 [14]. It is expected that as life expectancy rises, the cost of caring for older adults will increase, notably for the health care system [14]. In Bangladesh, stroke is more common in people aged >60 years. Stroke is Bangladesh’s third leading cause of death, behind coronary heart disease and infectious disease [12].

Several studies have been conducted to show the stroke prevalence in Bangladesh. In 2011, Mohammad et al [15] conducted a study on 15,627 individuals and reported the prevalence of stroke to be 3 per 1000 people. In 2015, Zaman et al [16] reported the prevalence of stroke to be 9.4 per 1000 people by taking a sample size of 1709 individuals. In 2018, Saha et al [17] conducted a study on 94,965 people and presented that the prevalence of stroke is 1.96 per 1000 people. In 2021, Mondal et al [18] reported the prevalence of stroke is 11.39 per 1000 people by taking a sample size of 25,287 individuals.

Stroke is influenced by various risk factors, with hypertension emerging as the predominant factor [19-21], closely followed by diabetes [19,21]. Hypertension is the main factor for stroke because it affects arteries throughout the body, resulting in more susceptibility to rupture or clogging. When the brain’s blood vessel is constricted or blocked by a clot, a portion of the brain no longer receives the necessary blood and oxygen, which causes stroke [22]. Furthermore, diabetes stops the body from properly digesting meals, causing the body to not produce or properly use insulin. It results in a buildup of glucose in the blood and damages blood arteries, which raise the risk of stroke [23]. In addition, some other risk factors are reported in several studies such as betel consumption [19], history of heart disease [24], dyslipidemia [25], metabolic syndrome [26], early atherosclerosis [21], smoking [21], and hypercholesterolemia [21].

Even though all these studies reported the prevalence and risk factors of stroke in Bangladesh, the sample size is not very large in terms of the population of Bangladesh, and data on stroke in rural areas are lacking. To create effective early prevention and intervention programs for stroke in Bangladesh, it is vital to comprehend the problem’s prevalence and its risk factors. So, the objectives of this study are to present the prevalence of stroke in a rural community in Bangladesh and to identify and associate various stroke risk factors.

## Methods

### Study Context

Bangladesh is a country in South Asia and is situated in the Bay of Bengal. It is divided into 8 administrative divisions [27]: Barisal, Chittagong, Dhaka, Khulna, Mymensingh, Rajshahi, Rangpur, and Sylhet. These divisions are further divided into districts, with a total of 64 districts in Bangladesh. Each district is then subdivided into subdistricts, and the subdistricts are further divided into unions, which consist of multiple villages. An overview of the administrative geography of Bangladesh is presented in Figure S1 in Multimedia Appendix 1.

### Study Design, Participants, and Data Collection Procedure

The research was carried out as part of the “Enriched Sastho” program, and the operation is currently operational in 7 divisions of Bangladesh. “Enriched Sastho” is a primary and preventive health care program created by Palli Karma Sahayak Foundation, Bangladesh. The program has 1 smartphone app for community health workers (CHWs) to collect data from rural people by visiting different households (Multimedia Appendix 1). During household visits, CHWs collect information such as sociodemographic, economic, environmental, and health-related information from individuals encompassing every age group and sex. Before collecting data, CHWs received 2 weeks of training to ensure the quality of the data collection and to familiarize themselves with the process.

For this investigation, we used data that were collected from July 2018 to June 2021. A total of 104 variables were obtained during data collection, encompassing sociodemographic, economic, environmental, and health-related information. This smartphone app is connected to Amazon Web Service (AWS) and all these data are usually stored in the AWS.

In this study, the presence of stroke was determined by survey questions asked by the CHWs to the individuals. The answer was categorized in binary format. Thus, if a person had a history of stroke, then the “stroke” variable was filled with 1, otherwise 0. In addition, CHWs checked their medical history and medical records to verify the stroke confirmation.

### Ethical Considerations

The United International University ethical review board authorized this study ( IREB/2023/008), and all participants gave written consent before participating in this study. Data were deidentified. As this protocol was part of a community-based health care model, no compensation was given to participants for taking part in the survey.

### Data Sampling Procedure

Initially, data were collected from a total of 1,352,724 individuals. A total of 4030 individuals were excluded due to missing birth dates, and 7088 individuals were excluded due to missing gender. Moreover, 17 individuals aged <18 years who had a stroke were excluded due to a lack of evidence of having a stroke. Following these exclusions, the remaining dataset consisted of a total of 1,341,589 individuals.

### Statistical Analysis

First, we collected data from AWS as a dump file. Then, we stored them in a MySQL server and extracted the required data from the original database by running MySQL queries. We examined the data for missing values and outliers. All the missing values and outlier-related instances were removed. We used descriptive statistics to assess the population’s age, gender, education, and occupation distribution. Numeric variables were represented by their mean and SD, while nominal variables were represented by their proportion. The prevalence of stroke per 1000 people was examined along with a 95% CI. We analyzed the prevalence of stroke by divisions to understand the stroke prevalence rate in different parts of Bangladesh.

In addition, the association of stroke risk predictors was calculated using multivariate logistic regression and presented in crude odds ratio (OR) and adjusted OR along with 95% CI. The risk predictors were: five socio-demographic variables (age, gender, education level, occupation, and union name) and 5 health-related comorbidities (stroke, diabetes, hypertension, cancer, CVD, and chronic obstructive pulmonary disease). Before using multivariate logistic regression, we checked for normality, multicollinearity, and outliers. The bar chart and radar chart were used to illustrate the major findings. All these analyses were done with the help of Python (Python Software Foundation) libraries like *pandas*, *Matplotlib*, *NumPy*, *SciPy*, *Plotly*, *stats model*, and *Seaborn*.

## Results

### Demographic Analysis

**Figure 1.**
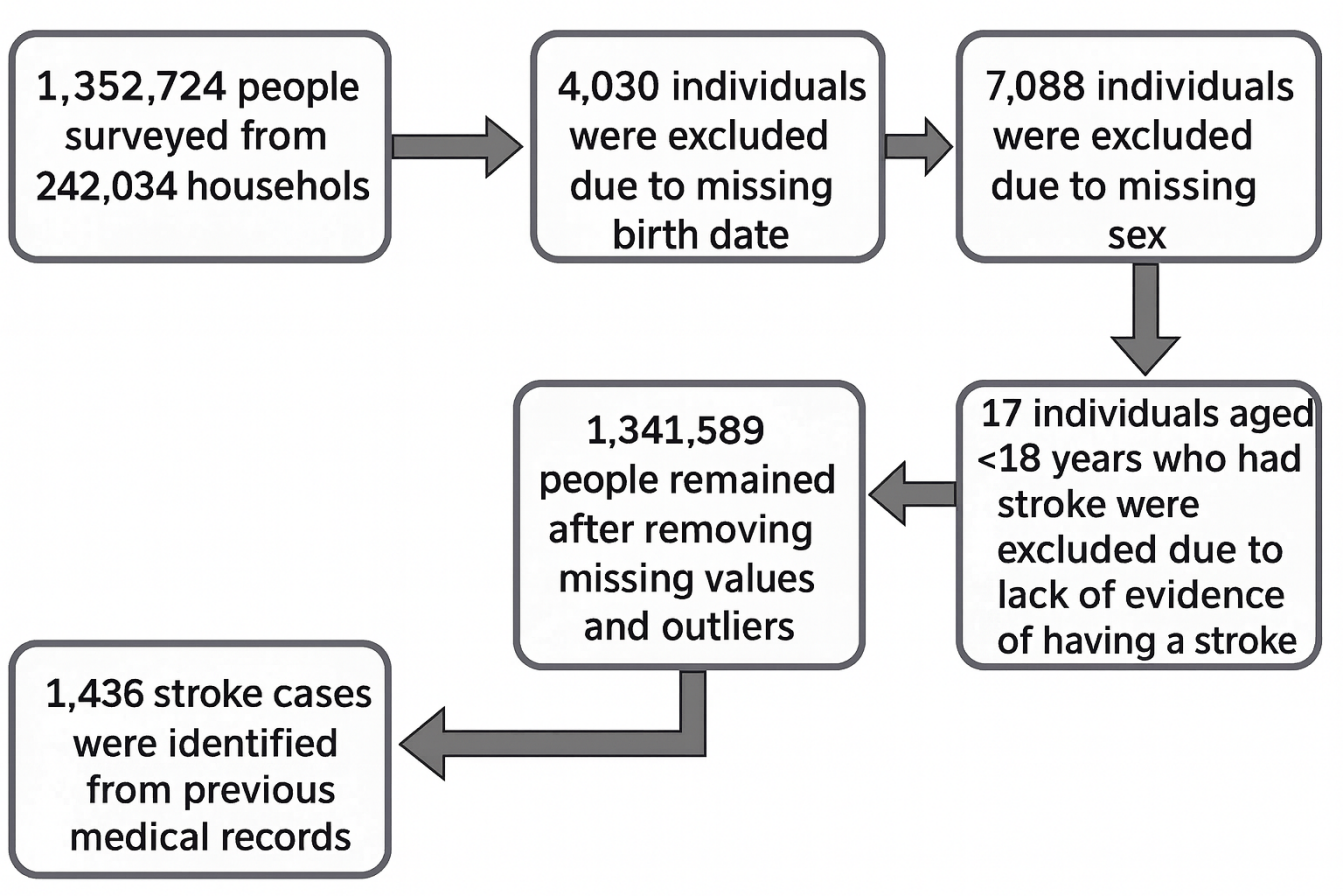
Sampling procedure overview.

An overview of the data sampling procedure is represented in Figure 1. From July 2018 to June 2021, the CHW questioned a total of 1,352,724 people from 242,034 households, encompassing 7 divisions. After removing missing values and outliers, a total of 1,341,589 people remained from the initial total population.

Detailed sociodemographic information is provided in Table 1. In our study, 51.33% (688,649/1,341,589) were male participants. The average (SD) age was 29.23 (19.05) years, with the average (SD) age of male participants being 29.57 (19.28) years and that of female participants being 28.87 (18.80) years. The majority of people (600,160/1,341,589, 44.74%) had an age of <25 years while the smallest proportion (10,865/1,341,589, 0.81%) of people had an age of ≥80 years. Besides, male participants were found in greater numbers than female participants among different age groups.

**Table 1.** Sociodemographic characteristics of study population (N=13,41,589).

Age (y)	Both sexes, n (%)	Male participants (n=688,649), n (%)	Female participants (n=652,940), n (%)
<25	600,160 (44.74)	304,148 (44.17)	296,012 (45.34)
25‐44	443,368 (33.05)	225,322 (32.72)	218,046 (33.39)
45‐54	138,896 (10.35)	73,573 (10.68)	65,323 (10)
55‐64	89,794 (6.69)	47,728 (6.93)	42,066 (6.44)
65‐79	58,506 (4.36)	32,314 (4.69)	26,192 (4.01)
≥80	10,865 (0.81)	5564 (0.81)	5301 (0.81)

#### Stroke Prevalence

There were 1436 stroke cases among 134,1589 people with an overall stroke prevalence rate of 1.07 (95% CI 1.02‐1.13) per 1000 people.

#### By Sex

Among 1436 patients with stroke, 894 were male participants and 542 were female participants. So, the stroke prevalence rate in male participants was 1.29 (95% CI 1.20–1.38) per 1000 people and that in female participants was 0.83 (95% CI 0.76‐0.90) per 1000 people. In other words, the stroke prevalence rate in male participants was 1.55 times higher than that in female participants.

#### By Age

**Figure 2.**
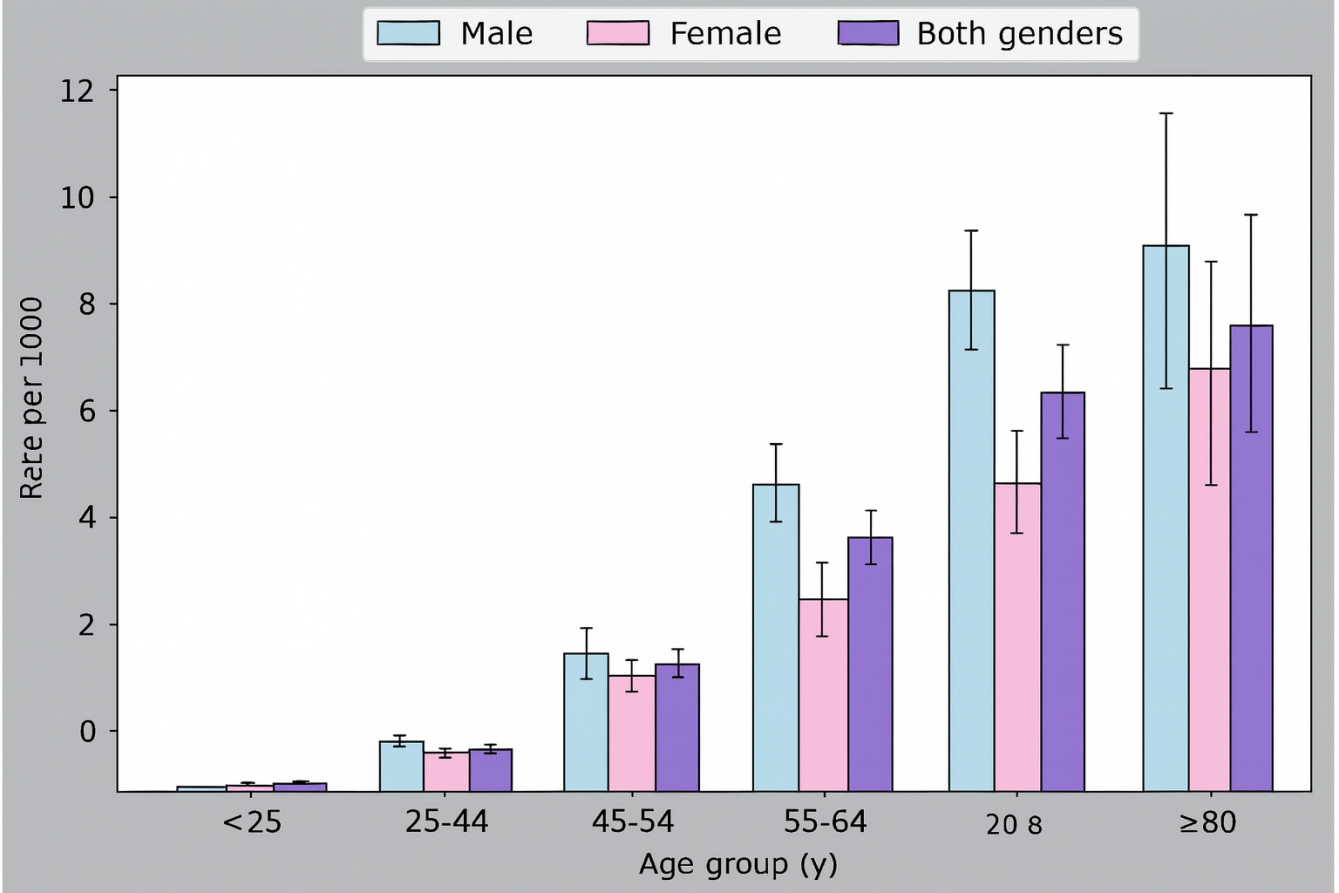
Prevalence of stroke per 1000 people with 95% CI.

Table 2 represents the distribution of the stroke population and their prevalence rates among different age groups. The overall stroke prevalence rate was high (8.84 per 1000) among the age group of “≥80” years, and the overall stroke prevalence rate was low (0.03 per 1000) among the age group of “<25” years. In addition, the overall stroke prevalence rate increased as age increased, and the stroke prevalence rate in male participants was higher than that in female participants among different age groups. This tendency is better depicted in Figure 2.

**Table 2.** Distribution of population with stroke and their prevalence rates among different age groups.

Age group (y)	Overall rate (%)^a^	95% CI	Rate in male participants (%)^a^	95% CI	Rate in female participants (%)^a^	95% CI
<25	17 (0.03)	0.01‐0.04	8 (0.03)	0.01‐0.04	9 (0.03)	0.01‐0.05
25‐44	197 (0.44)	0.38‐0.51	113 (0.5)	0.41‐0.59	84 (0.39)	0.3‐0.47
45‐54	284 (2.04)	1.81‐2.28	164 (2.23)	1.89‐2.57	120 (1.84)	1.51‐2.17
55‐64	398 (4.43)	4.0‐4.87	258 (5.41)	4.75‐6.06	140 (3.33)	2.78‐3.88
65‐79	444 (7.59)	6.89‐8.29	295 (9.13)	8.09‐10.17	149 (5.69)	4.78‐6.6
≥80	96 (8.84)	7.08‐10.6	56 (10.06)	7.44‐12.69	40 (7.55)	5.22‐9.88

a Prevalence rate per 1000 people.

#### By Area or Divisions of Bangladesh

**Figure 3.**
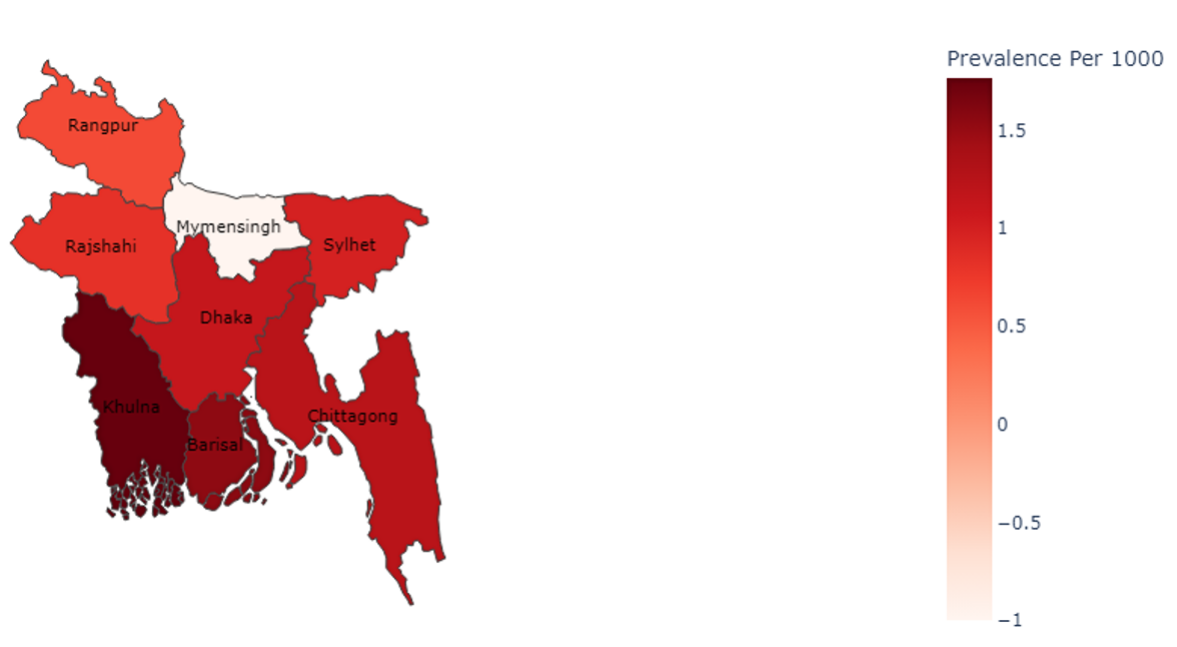
Division level heatmap representation of strokes prevalence in rural Bangladesh.

The prevalence of stroke by division is depicted in Table 3. In addition, Figure 3 displays a heatmap illustrating the prevalence rate of strokes at the division level in Bangladesh. The color scheme ranges from white, representing divisions with the lowest stroke prevalence, to the darkest red, indicating divisions with the highest stroke prevalence. In our study, we observed that the “Rangpur” division had the highest number of study participants, with a total of 273,718 people. On the other hand, the “Barisal” division had the lowest number of study participants, with a total of 52,330 people. In terms of stroke cases, the “Khulna” division had the highest number of cases, with a total of 370 stroke cases. Conversely, the “Barisal” division had the lowest number of stroke cases, with a total of 81 cases. It was also observed that the “Khulna” division (southwest of Bangladesh) had the highest stroke prevalence rate (1.77 per 1000 people) among all other 7 divisions (darkest red in Figure 3). The “Rangpur” division (northwest of Bangladesh) had the least stroke prevalence rate (0.62 per 1000 people; light red in Figure 3). In addition, detailed information on stroke prevalence at district and union levels is presented in Tables S1 and S2 in Multimedia Appendix 1.

**Table 3.** Prevalence of stroke by divisions.

Division	Stroke cases, n (%)	Total population	Prevalence per 1000
Barisal	81 (0.15)	52,330	1.55
Chittagong	312 (0.12)	252,239	1.24
Dhaka	165 (0.11)	146,200	1.13
Khulna	370 (0.18)	209,159	1.77
Rajshahi	200 (0.08)	240,801	0.83
Rangpur	169 (0.06)	273,718	0.62
Sylhet	139 (0.10%)	139,715	0.99

Table S3 in Multimedia Appendix 1 represents the prevalence of stroke by age groups for each division. From Table S3 in Multimedia Appendix 1, Barisal, Chittagong, Dhaka, Khulna, Rajshahi, Rangpur, and Sylhet had a total population of 52,330, 252,539, 146,200, 209,159, 240,801, 273,718, and 139,715, respectively. In the age group of ≥55 years, Barisal, Khulna, Chittagong, Rajshahi, Dhaka, Rangpur, and Sylhet had a total population of 6420, 26,158, 25,416, 32,946, 19,538, 35,077, and 13,610, respectively (Table S3 in Multimedia Appendix 1). These results confirmed that even though the Khulna division did not have more old people, stroke prevalence was still higher in the Khulna division.

### Stroke Risk Associations

Table 4 represents the distribution of patients with stroke having other noncommunicable diseases (NCDs). From Table 4, it was observed that, among patients with stroke, hypertension was the most prevalent among all NCDs. A total of 67.55% (95% CI 65.13‐69.97) of patients with stroke had hypertension. The second most prevalent was diabetes; 25.21% (95% CI 22.96‐27.45) of patients with stroke had diabetes. On the other hand, cancer was the least prevalent among patients with stroke, affecting only 0.63% (95% CI 0.22‐1.03). In addition, it is evident that female patients with stroke had a higher proportion of hypertension and diabetes compared to male patients with stroke. Conversely, male patients with stroke exhibited a higher proportion of cancer, CVD, and chronic obstructive pulmonary disease in comparison to female patients with stroke.

Table 5 represents the ORs of risk factors of stroke. From Table 5, it can be seen that individuals aged 65‐79 years were at a higher risk of having a stroke than other age groups (crude OR 9.883, 95% CI 8.834‐11.057, *P*<.05; adjusted OR 9.728 [adjusted for sex], 95% CI 8.694‐10.884*, P*<.05). In addition, male participants were at greater risk of having a stroke than female participants were (crude OR 1.565, 95% CI 1.406‐1.741, *P*<.05; adjusted OR 1.469 [adjusted for age], 95% CI 1.320‐1.636, *P*<.05). In addition, it can be recognized that, among hypertension, diabetes, and CVD, people who had CVD were at greater risk of having a stroke (crude OR 109.888, 95% CI 92.069‐131.156, *P*<.05; adjusted OR 40.232 [adjusted for age and sex], 95% CI 33.42‐48.437, *P*<.05). Besides, “Khulna” divisions (southwest of Bangladesh) people were at a greater risk of having a stroke (crude OR 1.881, 95% CI 1.671‐2.117, *P*<.05; adjusted OR 1.769 [adjusted for age and sex], 95% CI 1.571‐1.992, *P*<.05) than other division’s people.

**Table 4.** Distribution of patients with stroke having other noncommunicable diseases (NCDs; n=1436).

Disease	Overall		Male participants		Female participants	
	n (%)	95% CI	n (%)	95% CI	n (%)	95% CI
Diabetes	362 (25.21)	22.96‐27.45	221 (24.72)	21.89‐27.55	141 (26.01)	22.32‐29.71
Hypertension	970 (67.55)	65.13‐69.97	584 (65.32)	62.2‐68.44	386 (71.22)	67.41‐75.03
Cancer	9 (0.63)	0.22‐1.03	6 (0.67)	0.14‐1.21	3 (0.55)	0.07‐1.18
CVD^a^	150 (10.45)	8.86‐12.03	100 (11.19)	9.12‐13.25	50 (9.23)	6.79‐11.66
COPD^b^	113 (7.87)	6.48‐9.26	72 (8.05)	6.27‐9.84	41 (7.56)	5.34‐9.79

a CVD: cardiovascular disease.

b COPD: chronic obstructive pulmonary disease.

**Table 5.** Risk factors of stroke.

	*P* value (crude)	Crude odds ratio (95% CI)	*P* value (adjusted)	Adjusted odds ratio (95% CI)
Age (y)				
<45	<.05	0.050 (0.043‐0.58)	<.05	0.050 (0.044‐0.058)
45‐54	<.05	2.137 (1.877‐2.434)	<.05	2.120 (1.862‐2.415)
55‐64	<.05	5.365 (4.778‐6.023)	<.05	5.322 (4.740‐5.975)
65‐79	<.05	9.883 (8.834‐11.057)	<.05	9.728 (8.694‐10.884)
≥80	<.05	8.844 (7.183‐10.888)	<.05	8.852 (7.190‐10.898)
Sex				
Male	<.05	1.565 (1.406‐1.741)	<.05	1.469 (1.320‐1.636)
Female	N/A^a^	N/A	N/A	N/A
Disease				
Hypertension	<.05	80.642 (72.169‐90.108)	<.05	32.712 (28.977‐36.928)
Diabetes	<.05	31.733 (28.137‐35.789)	<.05	12.785 (11.31‐14.456)
CVD^b^	<.05	109.888 (92.069‐131.156)	<.05	40.232 (33.42‐48.437)
Division				
Chittagong	<.05	1.197 (1.056‐1.357)	<.05	1.415 (1.247‐1.605)
Rangpur	<.05	0.461 (0.393‐0.541)	<.05	0.480 (0.408‐0.563)
Rajshahi	<.05	0.739 (0.637‐0.859)	<.05	0.642 (0.553‐0.746)
Khulna	<.05	1.881 (1.671‐2.117)	<.05	1.769 (1.571‐1.992)
Dhaka	>.05	1.062 (0.903‐1.249)	>.05	0.943 (0.802‐1.110)
Sylhet	>.05	0.922 (0.774‐1.098)	>.05	1.062 (0.891‐1.265)
Barishal	<.05	1.474 (1.177‐1.844)	<.05	1.423 (1.136‐1.782)

a N/A: not applicable.

b CVD: cardiovascular disease.

## Discussion

### Principal Results

The main findings of our study were (1) the overall prevalence of stroke was 1.07 per 1000 people on analyzing 1.3 million individuals’ data, (2) the prevalence of stroke was the highest in the “Khulna” division (southwest of Bangladesh), and (3) hypertension was the major risk factor in patients with stroke. To the best of our knowledge, this study was the largest population-based investigation on the prevalence of strokes in rural Bangladesh.

### Comparison With Prior Work

From Table 6, it is evident that our findings have more similarity with those obtained by Saha et al [17] who conducted the study on 94,965 individuals and reported a prevalence of 1.9 per 1000 in the general population. We reported around a 14-fold higher sample size than that reported by Saha et al [17] and found a prevalence of 1.07 per 1000 in the general population. Other studies have calculated the prevalence on a much lower sample size as shown in Table 6. Moreover, it is noteworthy to mention that most of the studies have been performed in 1 specific region (Zaman et al [16] sampled data from only 1 rural area of “Chittagong” division; Saha et al [17] conducted a study from the data that were collected from only 1 rural area of “Rajshahi” division), whereas our study covered 7 divisions of Bangladesh.

**Table 6.** Comparison with other studies from Bangladesh.

Studies	Study setting	Age range (y)	Sample size	Prevalence rate per 1000
Mohammad et al [15]	Urban + rural	≥40	15,627	3
Zaman et al [16]	Rural	≥30	1709	9.4
Saha et al [17]	Rural	≥15	94,965	1.96
Mondal et al [18]	Urban + rural	≥18	25,287	11.39
This study	Rural		1,341,589	1.07
		<25	600,160	0.03
		25‐44	443,368	0.44
		45‐54	138,896	2.04
		55‐64	89,794	4.43
		65‐79	58,506	7.59
		≥80	10,865	8.84

Furthermore, this study was focused on rural settings. Mohammad et al [15] and Mondal et al [18] examined data from urban and rural settings and reported higher prevalence. However, these studies did not compare the prevalence between urban and rural settings. Therefore, we could not draw any conclusion from the fact that stroke prevalence may be higher in an urban setting than a rural setting. In addition, Mohammad et al [15] considered individuals aged 40 years and performed the study on 15,627 individuals. In our study of individuals aged ≥40 years, the prevalence was 3.42 per 1000 people, which is slightly higher than that observed in the study by Mohammad et al. Zaman et al [16] conducted a study on people aged ≥30 years on a sample size of 1709 and reported a prevalence of 9.4 per 1000. In our study, the prevalence was 2.23 per 1000 people for patients aged ≥30 years on a population of over 4,00,000. Here, it is noteworthy to mention that our sampled population had a higher proportion of younger people (44.74% of total individuals were aged <25 years) than other studies, which also could play a role in general prevalence calculation. Taken together, it is fair to assume that the lower prevalence reported in our study than in previous literature could be due to the following reasons: (1) the large sample size, (2) the vast region covered, (3) the rural setting, and (4) the proportion of the younger population.

We further found that male participants exhibited a considerably higher prevalence of stroke (1.55 times higher than that in female participants), similar to the findings of the previous study [17]. Moreover, we found that the stroke prevalence rate increased with age. This finding is also in line with the previous studies [12,17,18]. It is worth mentioning that we divided the age groups similarly to a previous study [17], and the age group ranges were different in previous studies [15,18].

Our division-based analysis showed that the highest stroke prevalence rate (1.77 per 1000 people) was observed in the “Khulna” division (southwest Bangladesh). This could be due to the high salt intake of the people since the “Khulna” division is one of the coastal areas of Bangladesh [28]. Islam et al [29] reported that 70% of groundwater in the “Khulna” district has a very high salinity hazard. In addition, several studies have associated a greater risk of developing hypertension with drinking high-salinity water in Bangladesh [30,31]. Furthermore, it is reported in other studies that the “Khulna” Division lacks primary and preventive health care facilities [32]. Due to this, the people of “Khulna” lack education on the disadvantages of drinking high-salinity water, smoking, and so on. Moreover, due to the lack of primary health care facilities, individuals may not seek regular medical consultations for ongoing monitoring of vital health indicators such as blood pressure and glucose levels. As a consequence, they may remain unaware of underlying conditions such as hypertension or diabetes, which are major risk factors of stroke. Taken together, we assumed that high salinity in water and lack of primary health care could be the main reasons for higher stroke prevalence in the “Khulna” division.

We found that hypertension (970/1436, 67.55%) and diabetes (362/1436, 25.21%) were the first and second most common risk factors of stroke than other NCDs. These findings are in line with the previous studies conducted in Bangladesh [17-19,22,23].

### Strengths and Limitations

This research has several strengths. First, this study is the largest sample size representation of the rural population of Bangladesh. In addition, we collected data from 50 unions representing rural settings all over Bangladesh. This study demonstrated the prevalence of stroke in 7 divisions of Bangladesh, which could be an accurate prevalence estimate of stroke. Second, we found that the stroke prevalence is higher in the “Khulna” division than in other divisions. It is a major finding in the context of Bangladesh, as the “Khulna” division has a high salt intake. We found that hypertension, diabetes, and other CVDs are leading risk factors for stroke in rural Bangladesh, which are known risk factors of stroke. Therefore, managing these diseases may help in managing stroke in rural Bangladesh.

However, this research has some limitations. Since all the data for this study were collected in a survey setting, the assessment of stroke with neurologists was not performed. However, to mitigate this challenge, the CHWs checked their medical records and medication history to make sure the information was accurate. However, we acknowledge the fact that the neurological assessment could lead to a more accurate prevalence estimation. Furthermore, we did not collect information regarding smoking, dietary habits, or family history of stroke. Future studies should include these variables to undertake better risk factor association studies.

### Conclusions

This study presented a comprehensive analysis of stroke prevalence in rural Bangladesh covering 7 divisions. One of our major findings was that the total prevalence rate was 1.07 per 1000 individuals in the general population with increasing prevalence in male participants and older populations. Although we reported lower stroke prevalence than in the contemporary studies, we believe this is due to the large sample size of our study. However, one major finding is that the southeast region of Bangladesh (“Khulna” division) has a higher prevalence than other divisions do. As the water of the “Khulna” division has a high salinity hazard along with a lack of primary health care facilities, our findings could help the policymakers to take necessary steps to mitigate these issues.

## Supplementary material

10.2196/46122 Multimedia Appendix 1 Supplementary document containing detailed results.

## Abbreviations

AWS: Amazon Web Service
CHW: community health worker
CVD: cardiovascular disease
DALY: disability-adjusted life year
NCD: noncommunicable disease
OR: odds ratio
