# Supplementary material for: Stroke Prevalence and Risk Factors in Rural Communities Within a Resource-Constrained South Asian Setting: Population-Based Study of 1.3 Million Individuals
Source: JMIR Public Health Surveill. 2025 Dec 19;11:e46122. doi: 10.2196/46122 (PMC12716629; doi:10.2196/46122)
Supplement: Multimedia Appendix 1 [file publichealth-v11-e46122-s001.docx]

**Supplementary File**

**S1: An overview of the administrative geography of Bangladesh:**


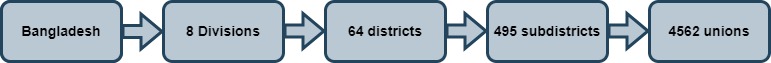


Fig S1: Administrative geography of Bangladesh

**S2: Enriched Sastho Smartphone Application:**

Community health workers (CHW) collect all the sociodemographic and healthcare-related data through the Enriched Sastho smartphone application. This application also features an offline syncing system. All the information gathered by CHW is first saved on the device's local system because rural locations frequently lack dependable internet access. Once the gadget establishes a dependable internet connection, all the data are then immediately uploaded to the cloud. Several important components of the Enriched Sastho smartphone application are shown in Fig. S2.


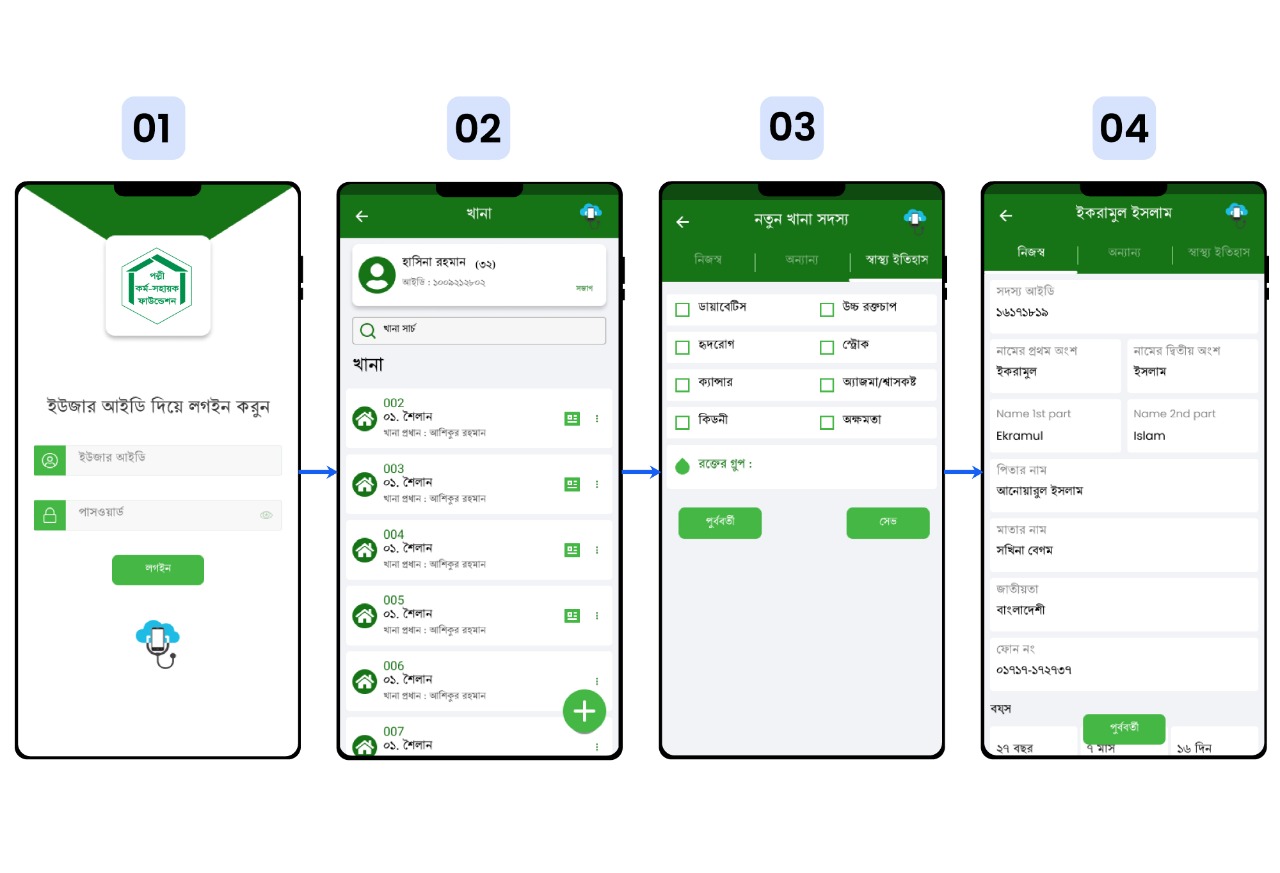


Fig S2: Enriched Sastho smartphone application.

**S3: Stroke prevalence by districts:**

We have collected data from a total of 34 districts in Bangladesh. From Table S1, it was observed that the ‘Khulna’ district had the highest stroke prevalence rate (4·57 per 1000 people) among all other 34 districts. The ‘Panchagarh’ district had the least stroke prevalence rate (0·04 per 1000 people). Fig S3 displays a heat map illustrating the prevalence rate of strokes at the district level in Bangladesh. The color scheme ranges from white, representing districts with the lowest stroke prevalence, to the darkest red, indicating districts with the highest stroke prevalence. According to Figure 3, the district with the highest stroke prevalence is “Khulna”, situated within the “Khulna” division, with a rate of 4.57 strokes per 1000 people. Moving on, the second highest stroke prevalence district is “Bagerhat”, located to the right of the “Khulna” district (as depicted in Figure S3).

Table S1: Prevalence of Stroke by Districts

| **Division** | **District** | **Stroke Cases** | **Total Population** | **Prevalence Per 1000** |
| --- | --- | --- | --- | --- |
| Khulna | Khulna | 91 | 19899 | 4·57 |
| Barisal | Jhalokati | 53 | 17681 | 3 |
| Khulna | Bagerhat | 79 | 27182 | 2·91 |
| Dhaka | Dhaka | 79 | 32827 | 2·41 |
| Chittagong | Comilla | 138 | 62206 | 2·22 |
| Sylhet | Maulvibazar | 62 | 28056 | 2·21 |
| Rajshahi | Rajshahi | 104 | 63484 | 1·64 |
| Dhaka | Gopalganj | 33 | 20378 | 1·62 |
| Khulna | Satkhira | 93 | 60286 | 1·54 |
| Rangpur | Gaibandha | 75 | 52208 | 1·44 |
| Chittagong | Chittagong | 137 | 97357 | 1·41 |
| Khulna | Meherpur | 51 | 36383 | 1·4 |
| Khulna | Chuadanga | 44 | 39870 | 1·1 |
| Sylhet | Sylhet | 75 | 76812 | 0·98 |
| Chittagong | Lakshmipur | 27 | 30388 | 0·89 |
| Barisal | Pirojpur | 17 | 19294 | 0·88 |
| Rangpur | Thakurgaon | 51 | 60472 | 0·84 |
| Rajshahi | Bogra | 32 | 40449 | 0·79 |
| Barisal | Bhola | 11 | 15355 | 0·72 |
| Dhaka | Manikganj | 20 | 28118 | 0·71 |
| Rajshahi | Naogaon | 51 | 79735 | 0·64 |
| Dhaka | Tangail | 21 | 36877 | 0·57 |
| Rangpur | Rangpur | 33 | 66323 | 0·5 |
| Khulna | Magura | 12 | 25539 | 0·47 |
| Dhaka | Madaripur | 12 | 28000 | 0·43 |
| Rajshahi | Joypurhat | 11 | 39837 | 0·28 |
| Rangpur | Dinajpur | 6 | 25540 | 0·23 |
| Chittagong | Cox's Bazar | 4 | 18497 | 0·22 |
| Chittagong | Rangamati | 4 | 20234 | 0·2 |
| Rajshahi | Pabna | 2 | 17296 | 0·12 |
| Chittagong | Noakhali | 2 | 23857 | 0·08 |
| Rangpur | Nilphamari | 3 | 44557 | 0·07 |
| Sylhet | Sunamganj | 2 | 34847 | 0·06 |
| Rangpur | Panchagarh | 1 | 24618 | 0·04 |

**
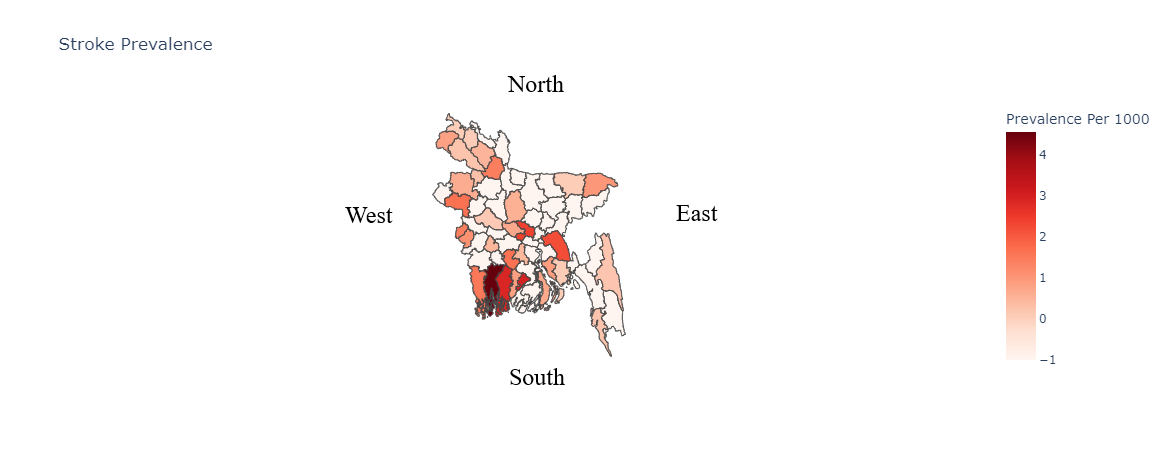
**

Fig S3: District-level heatmap representation of strokes prevalence in rural Bangladesh

**S4: Stroke prevalence by unions:**

We have collected data from a total of 50 unions in Bangladesh. Although stroke cases were observed in 49 unions. Table S2 showed that the ‘Bhandarpara’ union had the highest stroke prevalence rate (4·57 per 1000 people) among all other unions. The 'Debiduba' union had the least stroke prevalence rate (0·04 per 1000 people).

Table S2: Prevalence of Stroke by Unions

| **Union** | **Stroke Cases** | **Total Population** | **Prevalence Per 1000** |
| --- | --- | --- | --- |
| Bhandarpara | 91 | 19899 | 4·573094 |
| Charandwip | 61 | 18931 | 3·222228 |
| Kulkathi | 53 | 17681 | 2·997568 |
| Baruipara | 79 | 27182 | 2·906335 |
| Shibpur | 64 | 25512 | 2·508623 |
| Somvag | 79 | 32827 | 2·406556 |
| Majitpur | 79 | 34201 | 2·309874 |
| Panchgaon | 62 | 28056 | 2·209866 |
| Kamargaon | 64 | 30354 | 2·108454 |
| Eliotganj Dakkhin | 59 | 28005 | 2·106767 |
| Satkania | 62 | 30248 | 2·049722 |
| Sakhipur | 40 | 19856 | 2·014504 |
| Tetli | 47 | 27251 | 1·724707 |
| Andulbaria | 40 | 24102 | 1·659613 |
| Kushli | 33 | 20378 | 1·619393 |
| Gabtali Sadar | 31 | 19963 | 1·552873 |
| Tentulbaria | 51 | 36383 | 1·401754 |
| Atulia | 53 | 40430 | 1·310908 |
| Bilashbari | 35 | 26797 | 1·306116 |
| Sayedpur | 38 | 31359 | 1·211773 |
| Jahanabad | 40 | 33130 | 1·207365 |
| Omarpur | 23 | 25362 | 0·906869 |
| Laharkandi | 27 | 30388 | 0·888509 |
| Shikdermollik | 17 | 19294 | 0·881103 |
| Gongapur | 11 | 15355 | 0·716379 |
| Baniajuri | 20 | 28118 | 0·711288 |
| Golabari | 21 | 36877 | 0·569461 |
| Durgapur | 33 | 66323 | 0·497565 |
| Dhaneshwargati | 12 | 25539 | 0·46987 |
| Jabarhat Union | 13 | 29113 | 0·446536 |
| Khalia | 12 | 28000 | 0·428571 |
| Saghata | 11 | 26696 | 0·412047 |
| Cheragpur | 11 | 26889 | 0·409089 |
| Baratara | 9 | 22364 | 0·402432 |
| Mekhal | 13 | 34792 | 0·373649 |
| Uthali | 4 | 15768 | 0·253678 |
| Jotbani | 6 | 25540 | 0·234926 |
| Shilkhali | 4 | 18497 | 0·216251 |
| Hatkhola | 5 | 24199 | 0·20662 |
| Borkol | 4 | 20234 | 0·197687 |
| Kola | 5 | 26049 | 0·191946 |
| Chakla Union | 2 | 17296 | 0·115634 |
| Dhalahar | 2 | 17473 | 0·114462 |
| Char Amanullah | 2 | 23857 | 0·083833 |
| Nawapara | 1 | 13386 | 0·074705 |
| Botlagari | 3 | 44557 | 0·067329 |
| Surma | 2 | 34847 | 0·057394 |
| Elangi | 1 | 20486 | 0·048814 |
| Debiduba | 1 | 24618 | 0·040621 |

**S5: Stroke prevalence by age groups for each division:**

Table S3: Prevalence of stroke by age groups for each division

| **Barisal** | | | | **Khulna** | | | |
| --- | --- | --- | --- | --- | --- | --- | --- |
| **Age Groups** | **Stroke Cases** | **Total Population** | **Prevalence per 1000** | **Age Groups** | **Stroke Cases** | **Total Population** | **Prevalence per 1000** |
| <25 | 4 | 23270 | 0.171895 | <25 | 1 | 85104 | 0.01175 |
| 25-44 | 8 | 17202 | 0.465062 | 25-44 | 47 | 73285 | 0.641332 |
| 45-54 | 10 | 5438 | 1.838911 | 45-54 | 63 | 24612 | 2.559727 |
| 55-64 | 23 | 3501 | 6.569552 | 55-64 | 104 | 14855 | 7.00101 |
| 65-79 | 30 | 2496 | 12.01923 | 65-79 | 126 | 9540 | 13.20755 |
| >=80 | 6 | 423 | 14.1844 | >=80 | 29 | 1763 | 16.44923 |
| **Chittagong** | | | | **Rajshahi** | | | |
| **Age Groups** | **Stroke Cases** | **Total Population** | **Prevalence per 1000** | **Age Groups** | **Stroke Cases** | **Total Population** | **Prevalence per 1000** |
| <25 | 3 | 121692 | 0.024652 | <25 | 1 | 95307 | 0.010492 |
| 25-44 | 49 | 83490 | 0.586897 | 25-44 | 30 | 83416 | 0.359643 |
| 45-54 | 71 | 21941 | 3.235951 | 45-54 | 46 | 29132 | 1.57902 |
| 55-64 | 78 | 14154 | 5.51081 | 55-64 | 62 | 19210 | 3.227486 |
| 65-79 | 94 | 9607 | 9.784532 | 65-79 | 53 | 11635 | 4.555221 |
| >=80 | 17 | 1655 | 10.2719 | >=80 | 8 | 2101 | 3.807711 |
| **Dhaka** | | | | **Rangpur** | | | |
| **Age Groups** | **Stroke Cases** | **Total Population** | **Prevalence per 1000** | **Age Groups** | **Stroke Cases** | **Total Population** | **Prevalence per 1000** |
| <25 | 4 | 63475 | 0.063017 | <25 | 4 | 138603 | 0.028859 |
| 25-44 | 24 | 47400 | 0.506329 | 25-44 | 25 | 96814 | 0.258227 |
| 45-54 | 27 | 15787 | 1.710268 | 45-54 | 38 | 30351 | 1.252018 |
| 55-64 | 45 | 10578 | 4.254112 | 55-64 | 45 | 20010 | 2.248876 |
| 65-79 | 49 | 7424 | 6.600216 | 65-79 | 48 | 12877 | 3.727576 |
| >=80 | 16 | 1536 | 10.41667 | >=80 | 9 | 2190 | 4.109589 |
| **Sylhet** | | | |  | | | |
| **Age Groups** | **Stroke Cases** | **Total Population** | **Prevalence per 1000** |  |  |  |  |
| <25 | 0 | 72709 | 0 |  |  |  |  |
| 25-44 | 14 | 41761 | 0.335241 |  |  |  |  |
| 45-54 | 29 | 11635 | 2.49248 |  |  |  |  |
| 55-64 | 41 | 7486 | 5.47689 |  |  |  |  |
| 65-79 | 44 | 4927 | 8.930384 |  |  |  |  |
| >=80 | 11 | 1197 | 9.189641 |  |  |  |  |
